# Hydrophobic Coating of Paperboard Using Oak Wood-Derived Lignin Nanoparticles and Chitosan Composites

**DOI:** 10.3390/molecules30163387

**Published:** 2025-08-14

**Authors:** Le Van Hai, Eun Sol Heo, Cheol Yoon, Tae Hyun Kim

**Affiliations:** 1 Department of Materials Science and Chemical Engineering, Hanyang University, Ansan 15588, Gyeonggi-do, Republic of Korea; levanhai121978@gmail.com (L.V.H.); hes4584@hanyang.ac.kr (E.S.H.); cheolyoon@goodmac.kr (C.Y.); 2 Major in Advanced Materials and Semiconductor Engineering, School of Semiconductor Convergence Engineering, Hanyang University, Ansan 15588, Gyeonggi-do, Republic of Korea

**Keywords:** lignin, nanoparticles, chitosan, composites, green coating, water barrier

## Abstract

This study explores the potential application of lignin nanoparticles and chitosan–lignin nanoparticles (CLNs) as hydrophobic barrier coatings for paperboard. The lignin nanoparticles were initially prepared using a mixed solvent of ethanol and acetone. Their characteristics were examined via scanning electron microscopy (SEM) and dynamic light scattering, which revealed particle sizes in the range of 180–400 nm. The results indicated that the coatings with pure lignin nanoparticles failed to impart hydrophobicity to the paperboard, whereas the CLN coatings significantly enhanced hydrophobicity and reduced water absorption. The water contact angle increased from 109° to over 128° after the first CLN coating, remained at 127° with the second and third coating layers, and was maintained at 119° with four layers. Multilayer coatings were applied to improve barrier performance; however, no further enhancement in hydrophobicity was observed. The CLN-coated paper exhibited a significantly improved surface smoothness, as confirmed by SEM. The results indicate that a single-layer CLN coating is effective for imparting water-barrier properties to paperboard. In contrast, the coating with pure lignin nanoparticles resulted in cracked surfaces and inconsistent coating thicknesses.

## 1. Introduction

Lignin is the second most abundant bio-based organic polymer on Earth, after cellulose [1]. Lignin is typically obtained as a byproduct of cellulose production in the pulp and paper industry and is extracted from both wood and non-wood plants. While cellulose is commonly used in the production of pulp and paper, derivatives, textiles, and other products, most extracted lignin is treated as waste and used as fuel [2]. Lignin has long been considered a low-value-added product. Millions of tons of lignin are generated annually as waste from the kraft pulp industry [3,4,5]. Approximately 1–2% of lignin is converted into high-value-added products, whereas more than 90% is either burned for energy or simply discarded [5,6]. One of the major challenges in the utilization of lignin is its structural complexity. Lignin contains various phenolic groups and has a highly irregular and cross-linked structure that hinders its practical application. Additionally, different pretreatment methods and biomass sources result in variations in lignin structure, further complicating its industrial utilization [7].

Lignin can be extracted from lignocellulosic biomass through various methods, including alkaline, hydrothermal, and organosolv methods [8]. Alkaline solutions (e.g., NaOH, KOH, NH_4_OH) cleave ether and ester linkages in lignin–carbohydrate complexes, enhancing lignin solubility [9]. Hydrothermal treatment uses high-temperature water to hydrolyze the bond of lignin polymer [10]. In addition, organosolv extraction employs organic solvents with acid catalysts to break lignin linkages and separate it from other components [11]. Although acid treatments, such as those with H_2_SO_4_, HCl, and HNO_3_, are primarily used for carbohydrate hydrolysis, they may also cause partial lignin depolymerization under severe conditions [12]. The aforementioned lignin extraction methods are known to generate various lignins with different molecular weights, functional groups, and structural characteristics, which significantly influence the chemical reactivity and compatibility of lignin in downstream applications, particularly in the formation of lignin nanoparticles or polymer composites [13]. Therefore, the lignin extraction method is a critical step in determining the physical properties of biomass–polymer composite materials.

Zhao et al. [5] reported that lignin possesses multiple functional groups that form complex hydrogen-bonding networks, which can hinder its miscibility with polymer matrices and lead to interfacial compatibility issues in composite materials. Moreover, lignin exhibits a high degree of polydispersity in its molecular weight, which contributes to its heterogeneity, limiting its processability and reproducibility in advanced material applications [14]. However, lignin continues to attract interest because of its diverse functional groups, which can be modified for the development of fine chemicals and advanced materials. The 90% of lignin that is currently underutilized represents a significant resource for value-added applications.

Lignin has also attracted considerable attention owing to its diverse functionalities, including antioxidant activity, ultraviolet (UV)-shielding capability, nanocarrier formation, dye adsorption, microfluidic-device integration, surface coating, additive functionality, drug delivery, and antimicrobial properties [3,4,15,16,17]. Machado et al. [3] reported that lignin-based nanocarriers possess broad functionalities, including nutrient retention and the sustained release of herbicides and fungicides. Lignin has also been combined with chitosan for wastewater treatment and food packaging applications [16]. Yu et al. [17] reported the application of lignin/TiO_2_ composites for UV shielding in sunscreen products. The incorporation of lignin into TiO_2_ increased the sunscreen’s sun protection factor by approximately 30–60% compared with formulations containing only nano-TiO_2_. Recent studies on lignin valorization have explored its use as a precursor for carbon fibers and adhesives and as a sustainable alternative to petroleum-based chemicals [1,18].

The conversion of lignin into lignin nanoparticles is a research trend that explores the possibilities and potential future applications of lignin [19]. There are many methods for nanoparticle synthesis. Typically, lignin extracted from wood and non-wood materials is converted into lignin nanoparticles using solvents such as ethanol, methanol, acetone, ethylene glycol, and tetrahydrofuran [5]. Lignin nanoparticles offer improved dispersibility and reactivity compared with bulk lignin, making them suitable for nanocomposites, drug delivery, and functional coatings [20].

Chitosan is a bio-based material derived from chitin extracted from shrimp, lobster, and crab shells [21]. It is used for various applications, including wastewater treatment, dye removal, biomedical devices, and drug delivery [22,23,24]. Other applications of chitosan are food packaging, tissue engineering, scaffolding, and composite reinforcement, as reported elsewhere [25,26,27,28]. Chitosan has been combined with cellulose nanofibers, silver nanoparticles, and polypyrrole to improve the hydrophobicity of coated materials [29,30]. It has considerable potential for diverse applications in the environmental, biomedical, and composite fields [31]. Furthermore, the biocompatibility and biodegradability of chitosan have led to its approval for certain biomedical and food-related applications, highlighting its safety and versatility. However, the potential applications of chitosan require further investigation. In this study, chitosan–lignin composite nanoparticles (CLNs) were produced and used as a coating material on paperboard, and their effects on the hydrophobic properties of the paperboard were evaluated. In addition, lignin was investigated as a standalone coating material for paperboard because of its potential as a high-value-added product. The coating performance and hydrophobic properties of paperboard coated with lignin nanoparticles and CLNs were systematically investigated.

## 2. Results and Discussion

### 2.1. Lignin Nanoparticle Fabrication and Morphology

Lignin nanoparticles (LNPs) were fabricated using an ethanol/acetone solvent system. The fabricated lignin was dried in a vacuum oven at 45 °C for several days. The dried lignin was ground into a fine powder using a mortar and pestle and stored in sealed vials until use. The dried lignin powder was immersed overnight in ethanol/acetone under continuous stirring. The collected lignin nanoparticles were then used as a coating material.

**Figure 1:**
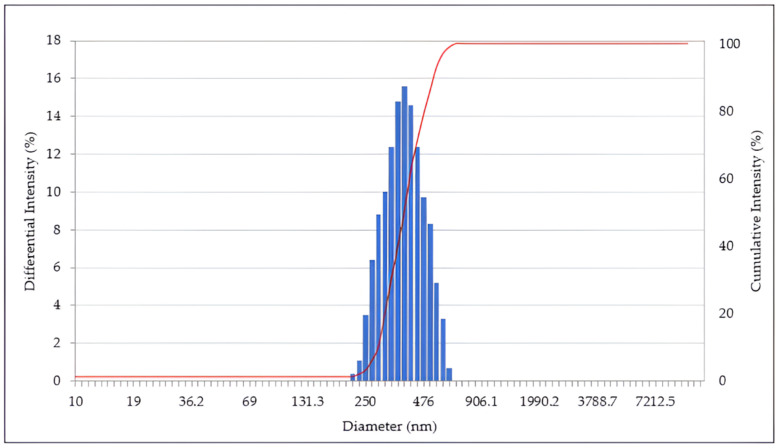
Size distribution of lignin nanoparticles analyzed via DLS. The blue columns indicate the differential intensity distribution, while the red line indicates the cumulative intensity distribution.

**Figure 2:**
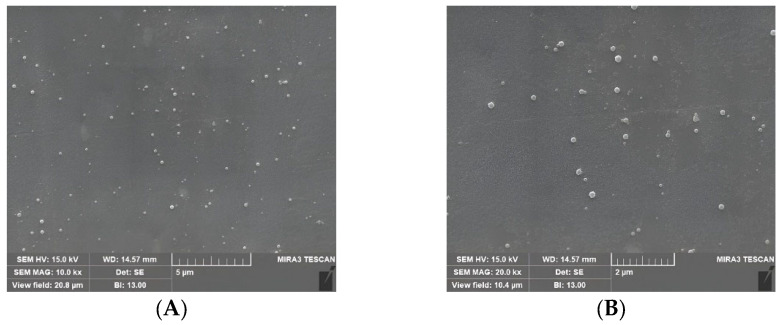
Lignin nanoparticles observed via SEM at magnifications corresponding to (**A**) 5 μm and (**B**) 2 μm scale bars.

Scanning electron microscopy (SEM) images indicated that the lignin nanoparticles had a spherical shape and uniform size. Figure 1 and Figure 2 show the particle size distribution determined via dynamic light scattering (DLS) and SEM images, respectively, of the LNPs.

### 2.2. DLS Spectrophotometer

The lignin nanoparticles were first analyzed using DLS, and the lignin particle size was found to be approximately 200–600 nm. The size distribution of the lignin nanoparticles obtained via DLS analysis is shown in Figure 1. The data analysis was performed at least four times to determine the particle size. The DLS analysis provided an overview of the lignin nanoparticle size distribution after fabrication. The largest cumulative number of lignin nanoparticles fell within the range of 300–400 nm, comprising approximately 70% of all nanoparticles. The remaining 30% of the nanoparticles were distributed in other size ranges: 15% were smaller than 300 nm, and another 15% were in the range of 500–650 nm. An example of a DLS intensity-based size distribution is shown in Figure 1.

### 2.3. SEM Analyzer

SEM was used to investigate the morphology of the lignin nanoparticles. The SEM images revealed the nanoparticles’ spherical shape. Furthermore, they confirmed that the size range of the nanoparticles was 200–400 nm, which was consistent with the DLS analysis results. No significant aggregation or clustering of nanoparticles was observed, suggesting effective stabilization during the fabrication process. Figure 2 shows the size and morphology of the fabricated lignin nanoparticles as observed via SEM.

### 2.4. Coatings of Lignin Nanoparticles and CLNs for Paper Products

**Figure 3:**
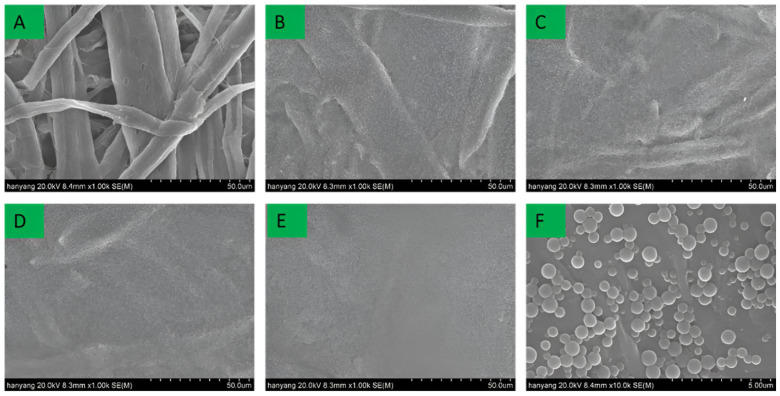
(**A**) Pristine paper substrate; (**B**–**E**) paper coated with chitosan–lignin nanoparticles in 1-, 2-, 3-, and 4-layer coatings, respectively (scale bar: 50 μm); (**F**) high-magnification image of the 1-layer chitosan–lignin nanoparticle coating (scale bar: 5 μm).

**Figure 4:**
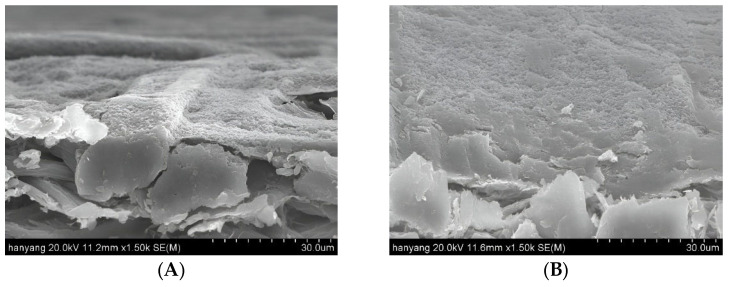
Cross-sectional SEM images of CLN-coated paperboard with (**A**) 1-layer and (**B**) 4-layer coatings (scale bar: 30 μm).

In this study, lignin nanoparticles and CLNs were investigated for their applicability as coating materials. Coatings with different basic weights were compared. Figure 3 and Figure 4 show SEM images of the surface and cross-section of the paperboard coated with CLNs.

The uncoated paperboard exhibited a highly textured surface with numerous voids, significant porosity, and a clearly defined cellulose fiber morphology. However, the surface of the paperboard became smoother with an increasing number of CLN coating layers. A single layer of CLNs formed a thin and discontinuous film on the paperboard surface (Figure 3B). After four CLN coating layers, almost no pores or cellulose fiber morphology was observed. Furthermore, no cracks or broken areas were observed on the surfaces of the coated paperboards. When coated with four layers, the paperboard exhibited a smooth surface, with a markedly different structure from that of the uncoated paperboard (Figure 3A). In contrast, with one to three coating layers, traces of the original cellulose fiber morphology remained partially visible. The lignin nanoparticles exhibited a spherical shape in SEM images, with particle sizes ranging from 200 to 500 nm, as confirmed by DLS and SEM. However, as shown in Figure 3F, the lignin nanoparticles in the coated paperboard formed aggregated clusters rather than maintaining individual dispersions. Figure 4 compares the cross-section of the one-layer and four-layer CLN coatings on the paperboard surface. The one-layer coating formed a thin and incomplete film, whereas the four-layer coating resulted in a significantly smoother and more continuous surface. This demonstrates that increasing the number of CLN layers improves the surface uniformity and coating quality.

**Figure 5:**
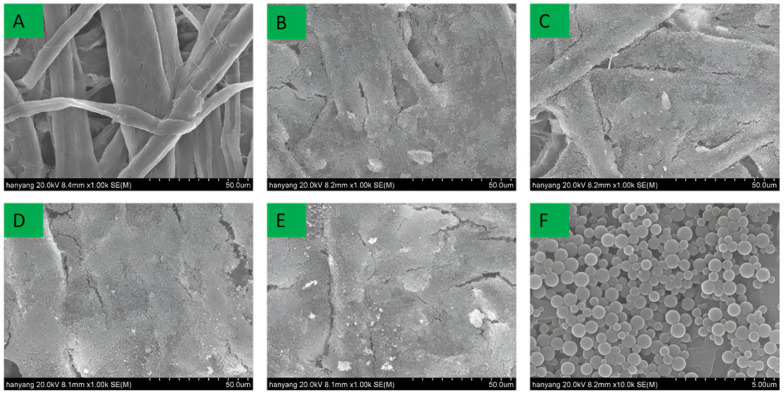
Surface morphology of (**A**) the uncoated paperboard substrate and (**B**–**E**) paperboard coated with lignin nanoparticles in 1-, 2-, 3-, and 4-layer coatings, respectively (scale bar: 50 μm); (**F**) high-magnification SEM image of 1-layer spray-coated lignin nanoparticles (scale bar: 5 μm).

**Figure 6:**
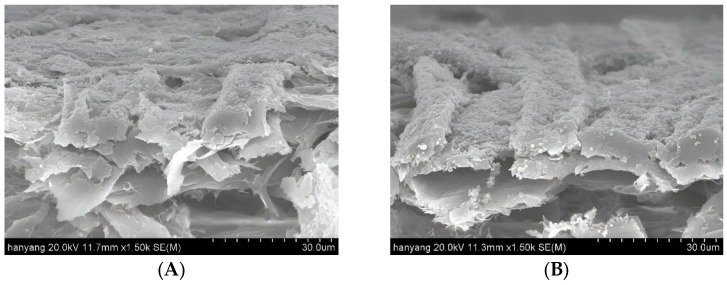
Cross-sectional SEM images of lignin nanoparticle-coated paperboard: (**A**) 1-layer and (**B**) 4-layer coatings (scale bar: 30 μm).

Figure 5 and Figure 6 show the effect of the pure lignin nanoparticle coating on paperboard. In the second method, only lignin nanoparticles were used to coat the paperboard surface. The pure lignin coating exhibited poor coating uniformity and a noncontinuous layer, in contrast to the uniform coating achieved with CLNs. The pure lignin nanoparticle coating exhibited cracks, along with poor continuity between coated regions. After three to four coating layers were applied, the cellulose fibers were no longer visible; however, cracks in the coating became more apparent. This indicated that the lignin nanoparticles were not well bonded or attached to the paper substrate and could easily be removed. In both the surface and cross-sectional views, the samples coated with pure lignin nanoparticles exhibited a rougher texture than did those coated with CLNs. These results demonstrate that the combination of CLNs provides better coating adhesion and surface smoothness on paper than pure lignin nanoparticle coatings. Figure 6 shows the cross-sectional differences between the one-layer and four-layer coatings using pure LNs. While the four-layer coating provided relatively improved surface coverage compared to the one-layer coating, the surface remained non-uniform and less smooth than that of the CLN-coated sample.

### 2.5. Water Contact Angles of Pure Paper and Coated Paper

**Figure 7:**
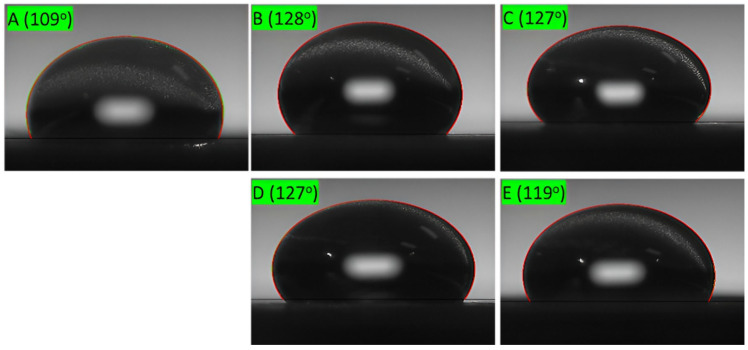
Surface morphology of (**A**) bare paperboard and paperboard coated with CLNs: (**B**) 1 layer; (**C**) 2 layers; (**D**) 3 layers; (**E**) 4 layers.

**Figure 8:**
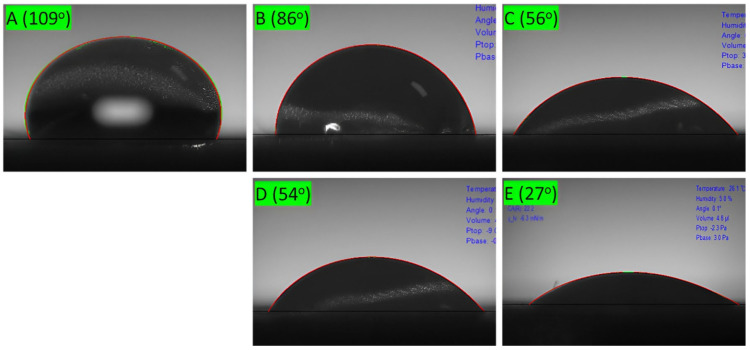
Surface morphology of (**A**) bare paperboard and lignin-coated paperboard with (**B)** 1 layer, (**C**) 2 layers, (**D**) 3 layers, and (**E**) 4 layers.

Figure 7 and Figure 8 show the water contact angles (WCAs) of the pure paperboard, paperboard coated with lignin nanoparticles, and CLN-coated paperboard with different numbers of coating layers. The pure paperboard exhibited a WCA of approximately 109°, indicating its inherent hydrophobicity. However, coatings of lignin nanoparticles and CLNs affected the surface hydrophobicity. The CLN coating significantly enhanced the hydrophobicity of the paperboard. The WCA increased from 109° for the pure paperboard to 128° and 127° with one to three CLN coating layers, but it decreased to 119° with four layers. These results suggest that single-layer CLN coatings have sufficient hydrophobicity for practical application. Increasing the number of coating layers did not significantly improve the hydrophobic coating performance. Further studies are needed to investigate the effects of the coating parameters and optimize the CLN coating process.

As shown in Figure 8, paperboard coated with pure lignin nanoparticles exhibited lower hydrophobicity than did paperboard coated with CLNs. The bare paperboard exhibited a WCA of 109°, which decreased as the number of pure lignin coating layers increased. The WCA decreased from 109° to 27° after four coating layers were applied. This suggests that pure lignin nanoparticles are ineffective at providing hydrophobicity. This indicates that pure lignin nanoparticles are not ideal for hydrophobic coating applications. Additionally, the surface coated with pure lignin nanoparticles exhibited cracks and discontinuous coverage, in contrast to the uniform film observed on the CLN-coated sample. The coated layer was easily detached from the paperboard after drying. This phenomenon was evident in the Cobb test. A visible water ring remained after each Cobb test regardless of whether one or more layers of lignin nanoparticles were applied. These results raise the question of which factors contribute to the superior hydrophobicity of CLN coatings. As discussed earlier, the CLN-coated paperboard exhibited a significant improvement, with the WCA increasing from 109° to 128°.

**Figure 9:**
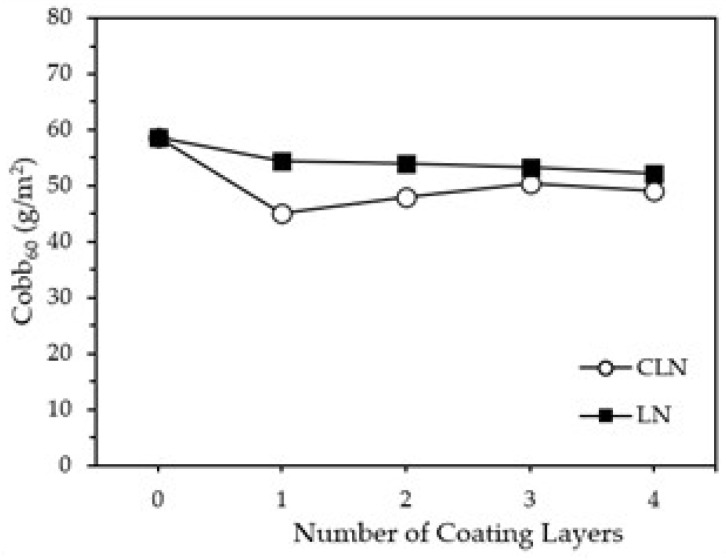
Cobb_60_ values of uncoated paperboard and paperboards coated with CLNs and LNs.

The Cobb_60_ test was performed to evaluate the hydrophobicity of coated paperboard samples, and the results are shown in Figure 9. Both LN- and CLN-coated paperboards exhibited lower Cobb_60_ values (52.2–54.5 g/m^2^ and 45.0–50.4 g/m^2^, respectively) than did the uncoated paperboard (58.6 g/m^2^), indicating enhanced hydrophobicity. For LN coatings, the Cobb_60_ values decreased from 54.5 g/m^2^ for the single-layered sample to 52.2 g/m^2^ for the four-layered sample as the number of coating layers increased. In contrast, the single-layer CLN coating exhibited a significantly decreased Cobb60 value (45.0 g/m^2^), then the value gradually increased again (47.9–49.1 g/m^2^). It is speculated that the single-layer CLN coating forms a relatively uniform and stable film, resulting in a notably reduced Cobb_60_ value. As the number of CLN layers increases, the effect of chitosan’s intrinsic hydrophilicity may become more pronounced, leading to a gradual increase in Cobb_60_ values. Although chitosan has both hydrophilic (hydroxyl group) and hydrophobic (acetyl group) sites, it has far more hydrophilic than hydrophobic sites; therefore, it is known to be a hydrophilic polymer.

### 2.6. Chemical Compositions of Chitosan, Lignin, and CLNs

**Figure 10:**
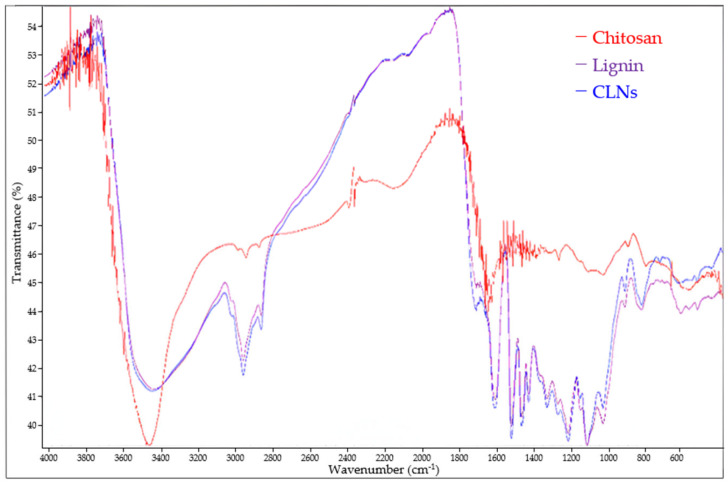
Fourier transform infrared (FT-IR) spectra of lignin nanoparticles, chitosan, and CLNs.

The chemical structures of chitosan, lignin, and the CLNs used for coating applications were investigated via FT-IR spectroscopy (Figure 10). A broad peak in the range of 3200–3450 cm^−1^ was assigned to OH stretching vibrations. Chitosan exhibited a sharper and more intense peak at approximately 3430 cm^−1^, whereas lignin and CLNs exhibited broader and less intense peaks in the range of 3300–3450 cm^−1^. The FT-IR spectrum also showed band shifts for chitosan at 2850, 2937, and 2970 cm^−1^ upon the formation of CLNs. These bands shifted toward the corresponding peaks of pure lignin with slightly lower intensities, whereas the broad O-H peak remained nearly unchanged. The peaks at 2937 and 2970 cm^−1^ were assigned to C-H stretching vibrations. The peak at approximately 1460 cm^−1^ was assigned to the C-H stretching of the aromatic skeleton, whereas the peak at approximately 1423 cm^−1^ corresponded to bending in the phenolic group [16,32].

## 3. Materials and Methods

### 3.1. Materials

#### 3.1.1. Feedstock

Oak wood was used in this study. It was cultivated and collected in Seoul, South Korea. After harvesting, it was air-dried at ambient temperature (25 °C) and then mechanically ground and passed through a sieve to obtain particles ranging from 500 to 2000 μm. The initial chemical composition, summarized in Table 1, was analyzed following the standard Laboratory Analytical Procedures (LAPs) (NREL/TP-510-42618 and NREL/TP-510-42622) [33,34]. 

#### 3.1.2. Chemicals

Low-molecular-weight chitosan was purchased from Sigma-Aldrich Inc. (Cat. No. 448869, Lot No. BCCD0403, St. Louis, MO, USA). Sodium hydroxide (NaOH; 97%, Cat. No. 367176, Sigma-Aldrich, St. Louis, MO, USA) was used for the biomass pretreatment. Sulfuric acid (H_2_SO_4_; 95–98%, ACS Reagent, Cat. No. 258105, Sigma-Aldrich, St. Louis, MO, USA), ethanol (ethanol, ≥99.9%, Cat. No. 4023–4110, Daejung Chemicals & Metals Co., Ltd., Siheung, Republic of Korea), and acetone (≥99.5%, Cat. No. 1009–4410, Daejung Chemicals & Metals Co., Ltd., Siheung, Republic of Korea) were used without further purification.

### 3.2. Lignin Extraction and Purification

Lignin was isolated from oak wood via treatment with 5.0% sodium hydroxide at 180 °C for 2 h. The resulting black liquor was filtered through glass filters to eliminate the remaining cellulose fibers and larger particles. Sulfuric acid was added to the black liquor to adjust the pH to approximately 2.5, inducing lignin precipitation. The precipitated solution was collected after settling overnight. The precipitate was washed multiple times via centrifugation at 10,000 rpm for 10–20 min using a centrifuge (Combi 514R, Hanil Scientific Inc., Gimpo, Republic of Korea) until the solution reached a pH of approximately 6. After washing, the lignin was dried at 45 °C in an oven for several days until it reached a constant weight, ground into a fine powder, and stored in a glass container until use. Lignin nanoparticles were synthesized using a solvent-exchange technique. The morphology, chemical structure, and coating performance of both the raw lignin and the synthesized nanoparticles were analyzed. Coating layers composed of either lignin nanoparticles alone or CLNs were prepared and applied as one to four sequential layers.

### 3.3. Lignin Nanoparticle Fabrication

The ground lignin particles were dissolved in an ethanol/acetone solvent overnight at room temperature. Subsequently, deionized (DI) water was added for approximately 15 min, and the volume of water required to dissolve the lignin solvent was 1:5 or 1:6 for obtaining the lignin nanoparticles. In brief, each batch of lignin nanoparticles was prepared by dissolving 2.0 g of the extracted lignin in 95% ethanol solvent under magnetic stirring. The samples were mixed at 200–300 RPM overnight. After that the LNs were precipitated overnight or collected by centrifugation. The suspension was then transferred to an evaporation dryer to evaporate the ethanol at 45 °C for 72 h until a constant weight was reached. The lignin nanoparticles were stored in a refrigerator until further use. The remaining lignin after ethanol treatment was further treated with acetone. In this step, 20 mL of acetone was added to the undissolved lignin and mixed at 200–300 RPM for approximately one hour. After that, DI was added and was stirred overnight. The LNs treated with acetone solvent were collected by centrifugation, and the acetone was removed using a rotary evaporator (RV 8 S99, IKA, Staufen, Germany) equipped with a heating bath (HB 10 S99).

### 3.4. Coating Method

Coating was performed using an autofilm applicator (DA-QP100, Dong Ah Trade Corp, Seoul, Republic of Korea). Lignin nanoparticles and chitosan were weighed for a 95:5 combination. Defined amounts of CLNs with 3% consistency were used for one-face coating application. The coating was applied using a bar coater (RDS26, Dong Ah Trade Corp, Seoul, Republic of Korea) at a speed of 50 mm/s. For comparison, the same coating procedure was also conducted using lignin nanoparticles alone, without chitosan. The coating weight depended on the number of layers, ranging from 1.67 g/m^2^ for a single layer to 6.67 g/m^2^ for four layers. The coated samples were dried in an oven at 105 °C for 10–15 min after the deposition of each layer, and this process was continued until the final layer was applied. After the coating process, the sample was kept in the oven dryer at 105 °C for approximately 3 h before being used for the Cobb_60_ or WCA test.

### 3.5. Lignin Morphology

The particle size was analyzed via DLS (Photal, Otsuka Electronics Co., Ltd., Osaka, Japan). Measurements were performed at 25 °C using DI water as a diluent. The refractive index and dynamic viscosity of the medium were measured as 1.3328 and 0.9 mPa·s, respectively. More than four replicates of the lignin nanoparticles were used. Thus, the lignin nanoparticle size distribution was determined.

SEM (S-900, Hitachi Co., Tokyo, Japan) was used to evaluate the morphology of the lignin nanoparticles. A diluted solution of lignin nanoparticles was prepared, and a drop of the diluted solution was placed on Ag tape attached to the SEM holder. After drying, the samples were analyzed using SEM.

### 3.6. FT-IR Analysis of CLNs

The FT-IR spectra of chitosan, lignin, and CLNs were measured using a FT-IR spectrometer (Nicolet IS10, Thermo Scientific, Waltham, MA, USA) equipped with an attenuated total reflectance accessory. Each sample was analyzed in the wavenumber range of 650–4000 cm^−1^ to identify the presence of characteristic functional groups and observe possible changes in chemical structure resulting from nanoparticle formation. To ensure reliable measurements, each spectrum was collected by averaging multiple scans per sample.

### 3.7. Hydrophobicity of Lignin Nanoparticles and Lignin/Chitosan Composite

The hydrophobicity of the lignin and lignin nanoparticle-coated paper was evaluated by measuring their WCAs. The WCAs were measured using an optical contact-angle analyzer (SDL200TEZD, FEMTOFAB Co., Ltd., Pohang, Republic of Korea). For the WCA analysis, a 5 μL drop of deionized water was deposited on the surface of the paper or coated paper, and the contact angle was measured shortly after droplet placement. The measurement angles were obtained by averaging the contact angle values measured at 10 different locations on each sample. This procedure helped minimize variations due to surface irregularities and ensured consistent evaluation across different samples.

## 4. Conclusions

Lignin was extracted from oak wood using NaOH and converted into lignin nanoparticles using an ethanol/acetone solvent. The lignin nanoparticles had sizes of 200–400 nm, as determined via DLS and SEM. They were combined with chitosan to prepare CLNs, which were applied to paper as a hydrophobic coating. The paper coated with lignin nanoparticles alone did not exhibit hydrophobic properties. In comparison, the CLNs exhibited superior hydrophobic properties, along with substantially improved hydrophobicity, with a WCA of 128° compared to 109° for pure paperboard. One to three layers of coated CLNs would be a good solution, as the hydrophobicity decreased with additional coating cycles. In this study, the detailed mechanism and composition optimization of the CLNs were not investigated. These are important topics for future work. In future research, we plan to study methods to increase the biomass content of coating materials and improve properties such as hydrophobicity, compatibility with polymers, and gas barrier properties. Based on this material, we will also focus on finding methods to utilize CLNs for packaging material applications.

## Figures and Tables

**Table 1:** Solid-composition analysis of oak wood.

Component	Composition [%]
Glucan	38.6 ± 1.6
Xylan	6.2 ± 0.1
Galactan	trace
Arabinan	2.3 ± 0.2
Mannan	12.6 ± 1.5
ASL	5.6 ± 0.5
AIL	27.5 ± 1.2
Ash	0.6 ± 0.1
Total	93.4

Notes: All weight percentages were calculated using the oven-dry weight biomass. The data in the table represent the mean (*n* = 3) ± standard deviation. ASL: acid-soluble lignin, AIL: acid-insoluble lignin.

## Data Availability

The data supporting the findings of this study are available within the article.
